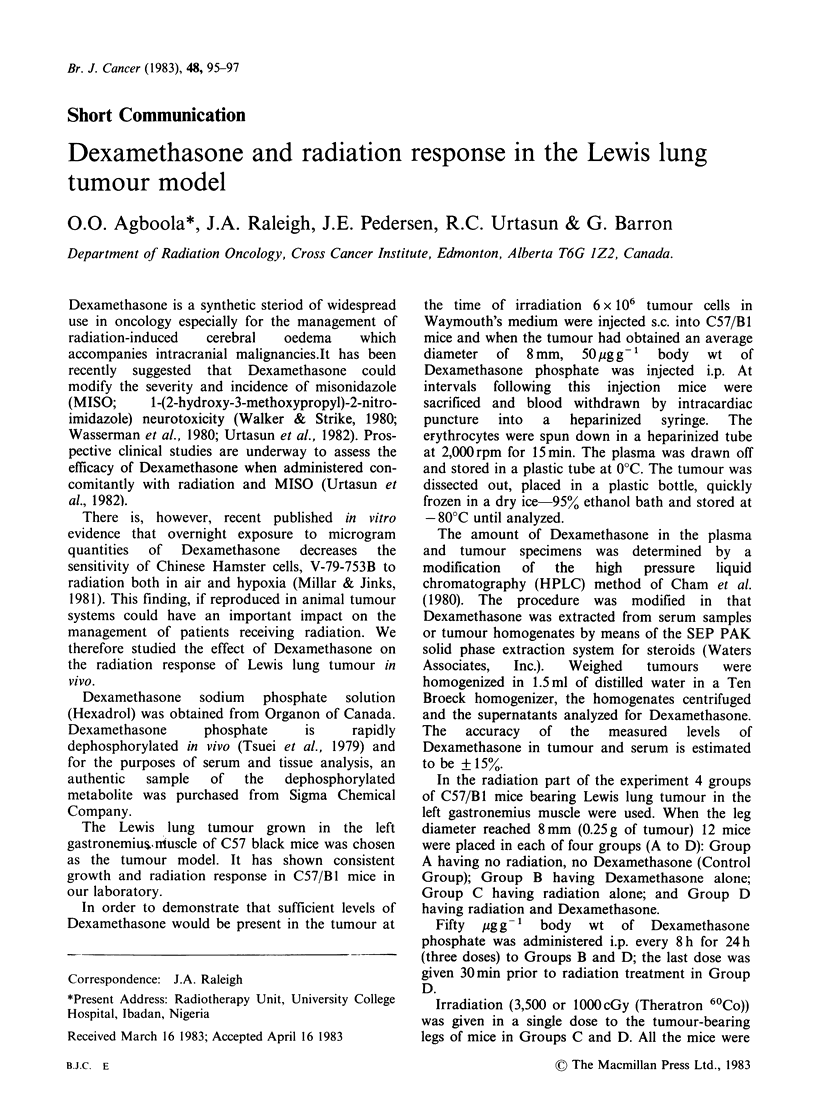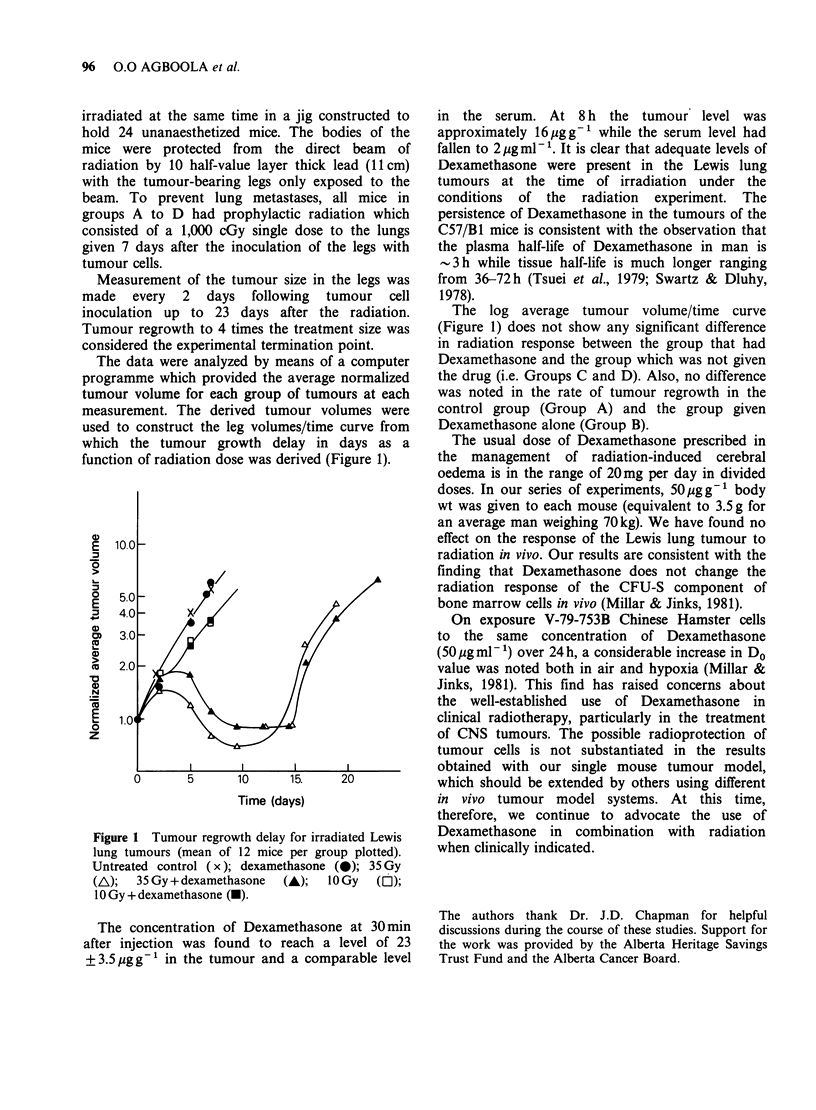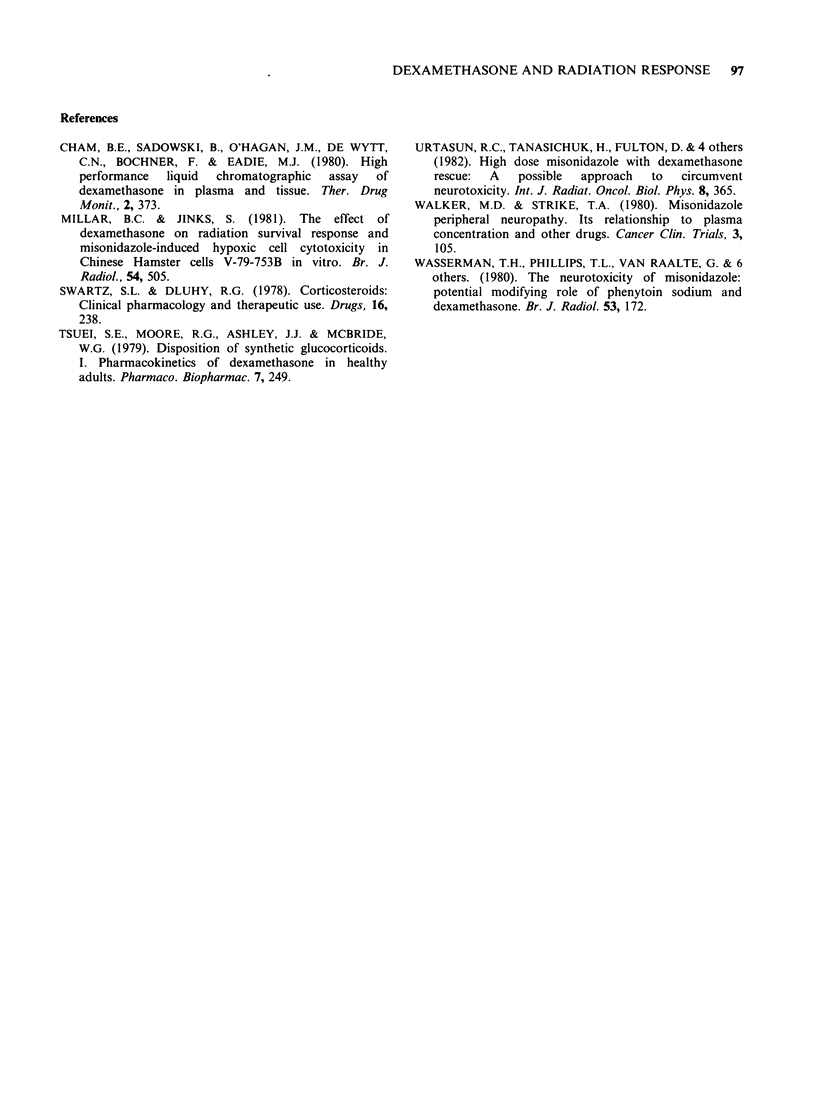# Dexamethasone and radiation response in the Lewis lung tumour model.

**DOI:** 10.1038/bjc.1983.162

**Published:** 1983-07

**Authors:** O. O. Agboola, J. A. Raleigh, J. E. Pedersen, R. C. Urtasun, G. Barron


*Br. J. Cancer* (1983), **48**, 95–97

### Short Communication

### Dexamethasone and radiation response in the Lewis lung tumour model

O.O. Agboola*, J.A. Raleigh, J.E. Pedersen, R.C. Urtasun & G. Barron

*Department of Radiation Oncology, Cross Cancer Institute, Edmonton, Alberta T6G 1Z2, Canada.*

Dexamethasone is a synthetic steriod of widespread use in oncology especially for the management of radiation-induced cerebral oedema which accompanies intracranial malignancies.It has been recently suggested that Dexamethasone could modify the severity and incidence of misonidazole (MISO; 1-(2-hydroxy-3-methoxypropyl)-2-nitro-imidazole) neurotoxicity (Walker & Strike, 1980; Wasserman *et al.*, 1980; Urtasun *et al.*, 1982). Prospective clinical studies are underway to assess the efficacy of Dexamethasone when administered concomitantly with radiation and MISO (Urtasun *et al.*, 1982).

There is, however, recent published *in vitro* evidence that overnight exposure to microgram quantities of Dexamethasone decreases the sensitivity of Chinese Hamster cells, V-79-753B to radiation both in air and hypoxia (Millar & Jinks, 1981). This finding, if reproduced in animal tumour systems could have an important impact on the management of patients receiving radiation. We therefore studied the effect of Dexamethasone on the radiation response of Lewis lung tumour *in vivo*.

Dexamethasone sodium phosphate solution (Hexadrol) was obtained from Organon of Canada. Dexamethasone phosphate is rapidly dephosphorylated *in vivo* (Tsuei *et al.*, 1979) and for the purposes of serum and tissue analysis, an authentic sample of the dephosphorylated metabolite was purchased from Sigma Chemical Company.

The Lewis lung tumour grown in the left gastronemius muscle of C57 black mice was chosen as the tumour model. It has shown consistent growth and radiation response in C57/B1 mice in our laboratory.

In order to demonstrate that sufficient levels of Dexamethasone would be present in the tumour at the time of irradiation $6\times10^6$ tumour cells in Waymouth's medium were injected s.c. into C57/B1 mice and when the tumour had obtained an average diameter of 8 mm, $50\,\mu g\,g^{-1}$ body wt of Dexamethasone phosphate was injected i.p. At intervals following this injection mice were sacrificed and blood withdrawn by intracardiac puncture into a heparinized syringe. The erythrocytes were spun down in a heparinized tube at 2,000 rpm for 15 min. The plasma was drawn off and stored in a plastic tube at 0°C. The tumour was dissected out, placed in a plastic bottle, quickly frozen in a dry ice—95% ethanol bath and stored at −80°C until analyzed.

The amount of Dexamethasone in the plasma and tumour specimens was determined by a modification of the high pressure liquid chromatography (HPLC) method of Cham *et al.* (1980). The procedure was modified in that Dexamethasone was extracted from serum samples or tumour homogenates by means of the SEP PAK solid phase extraction system for steroids (Waters Associates, Inc.). Weighed tumours were homogenized in 1.5 ml of distilled water in a Ten Broeck homogenizer, the homogenates centrifuged and the supernatants analyzed for Dexamethasone. The accuracy of the measured levels of Dexamethasone in tumour and serum is estimated to be ±15%.

In the radiation part of the experiment 4 groups of C57/B1 mice bearing Lewis lung tumour in the left gastronemius muscle were used. When the leg diameter reached 8 mm (0.25 g of tumour) 12 mice were placed in each of four groups (A to D): Group A having no radiation, no Dexamethasone (Control Group); Group B having Dexamethasone alone; Group C having radiation alone; and Group D having radiation and Dexamethasone.

Fifty $\mu g\,g^{-1}$ body wt of Dexamethasone phosphate was administered i.p. every 8 h for 24 h (three doses) to Groups B and D; the last dose was given 30 min prior to radiation treatment in Group D.

Irradiation (3,500 or 1000 cGy (Theratron $^{60}$Co)) was given in a single dose to the tumour-bearing legs of mice in Groups C and D. All the mice were

Correspondence: J.A. Raleigh

*Present Address: Radiotherapy Unit, University College Hospital, Ibadan, Nigeria

Received March 16 1983; Accepted April 16 1983

© The Macmillan Press Ltd., 1983

irradiated at the same time in a jig constructed to hold 24 unanaesthetized mice. The bodies of the mice were protected from the direct beam of radiation by 10 half-value layer thick lead (11 cm) with the tumour-bearing legs only exposed to the beam. To prevent lung metastases, all mice in groups A to D had prophylactic radiation which consisted of a 1,000 cGy single dose to the lungs given 7 days after the inoculation of the legs with tumour cells.

Measurement of the tumour size in the legs was made every 2 days following tumour cell inoculation up to 23 days after the radiation. Tumour regrowth to 4 times the treatment size was considered the experimental termination point.

The data were analyzed by means of a computer programme which provided the average normalized tumour volume for each group of tumours at each measurement. The derived tumour volumes were used to construct the leg volumes/time curve from which the tumour growth delay in days as a function of radiation dose was derived (Figure 1).

**Figure 1** Tumour regrowth delay for irradiated Lewis lung tumours (mean of 12 mice per group plotted). Untreated control (×); dexamethasone (●); 35 Gy (△); 35 Gy + dexamethasone (▲); 10 Gy (□); 10 Gy + dexamethasone (■).

The concentration of Dexamethasone at 30 min after injection was found to reach a level of 23 $\pm 3.5\,\mu g\,g^{-1}$ in the tumour and a comparable level in the serum. At 8 h the tumour level was approximately $16\,\mu g\,g^{-1}$ while the serum level had fallen to $2\,\mu g\,ml^{-1}$. It is clear that adequate levels of Dexamethasone were present in the Lewis lung tumours at the time of irradiation under the conditions of the radiation experiment. The persistence of Dexamethasone in the tumours of the C57/B1 mice is consistent with the observation that the plasma half-life of Dexamethasone in man is ~3 h while tissue half-life is much longer ranging from 36–72 h (Tsuei *et al.*, 1979; Swartz & Dluhy, 1978).

The log average tumour volume/time curve (Figure 1) does not show any significant difference in radiation response between the group that had Dexamethasone and the group which was not given the drug (i.e. Groups C and D). Also, no difference was noted in the rate of tumour regrowth in the control group (Group A) and the group given Dexamethasone alone (Group B).

The usual dose of Dexamethasone prescribed in the management of radiation-induced cerebral oedema is in the range of 20 mg per day in divided doses. In our series of experiments, $50\,\mu g\,g^{-1}$ body wt was given to each mouse (equivalent to 3.5 g for an average man weighing 70 kg). We have found no effect on the response of the Lewis lung tumour to radiation *in vivo*. Our results are consistent with the finding that Dexamethasone does not change the radiation response of the CFU-S component of bone marrow cells *in vivo* (Millar & Jinks, 1981).

On exposure V-79-753B Chinese Hamster cells to the same concentration of Dexamethasone ($50\,\mu g\,ml^{-1}$) over 24 h, a considerable increase in $D_0$ value was noted both in air and hypoxia (Millar & Jinks, 1981). This find has raised concerns about the well-established use of Dexamethasone in clinical radiotherapy, particularly in the treatment of CNS tumours. The possible radioprotection of tumour cells is not substantiated in the results obtained with our single mouse tumour model, which should be extended by others using different *in vivo* tumour model systems. At this time, therefore, we continue to advocate the use of Dexamethasone in combination with radiation when clinically indicated.

The authors thank Dr. J.D. Chapman for helpful discussions during the course of these studies. Support for the work was provided by the Alberta Heritage Savings Trust Fund and the Alberta Cancer Board.

### References

CHAM, B.E., SADOWSKI, B., O'HAGAN, J.M., DE WYTT, C.N., BOCHNER, F. & EADIE, M.J. (1980). High performance liquid chromatographic assay of dexamethasone in plasma and tissue. *Ther. Drug Monit.*, **2,** 373.

MILLAR, B.C. & JINKS, S. (1981). The effect of dexamethasone on radiation survival response and misonidazole-induced hypoxic cell cytotoxicity in Chinese Hamster cells V-79-753B in vitro. *Br. J. Radiol.*, **54,** 505.

SWARTZ, S.L. & DLUHY, R.G. (1978). Corticosteroids: Clinical pharmacology and therapeutic use. *Drugs,* **16,** 238.

TSUEI, S.E., MOORE, R.G., ASHLEY, J.J. & MCBRIDE, W.G. (1979). Disposition of synthetic glucocorticoids. I. Pharmacokinetics of dexamethasone in healthy adults. *Pharmaco. Biopharmac.* **7,** 249.

URTASUN, R.C., TANASICHUK, H., FULTON, D. & 4 others (1982). High dose misonidazole with dexamethasone rescue: A possible approach to circumvent neurotoxicity. *Int. J. Radiat. Oncol. Biol. Phys.* **8,** 365.

WALKER, M.D. & STRIKE, T.A. (1980). Misonidazole peripheral neuropathy. Its relationship to plasma concentration and other drugs. *Cancer Clin. Trials,* **3,** 105.

WASSERMAN, T.H., PHILLIPS, T.L., VAN RAALTE, G. & 6 others. (1980). The neurotoxicity of misonidazole: potential modifying role of phenytoin sodium and dexamethasone. *Br. J. Radiol.* **53,** 172.